# New insight into the role of NT-proBNP in alcoholic liver cirrhosis as a noninvasive marker of esophageal varices

**DOI:** 10.3325/cmj.2012.53.374

**Published:** 2012-08

**Authors:** Neven Ljubičić, Marija Gomerčić, Dražen Zekanović, Tomislava Bodrožić-, Džakić, Ana Đuzel

**Affiliations:** 1 Division of Gastroenterology, Department of Internal Medicine, University Hospital Sestre Milosrdnice, University of Zagreb School of Medicine, Zagreb, Croatia; 2 Department of Internal Medicine, General Hospital Zadar, Zadar, Croatia

## Abstract

**Aim:**

To investigate the association between plasma concentrations of N-terminal pro-B-type natriuretic peptide (NT-proBNP) and formation of esophageal varices.

**Methods:**

Thirty-five patients with alcoholic cirrhosis were divided into three groups according to the Child-Pugh classification: grade A (n = 11, 32%), B (n = 12, 34%), and C (n = 12, 34%). System hemodynamic parameters were measured using sphygmomanometry, electrocardiography, and echocardiography. NT-proBNP was analyzed by using an electrochemiluminiscence sandwich immunoassay.

**Results:**

The presence of esophageal varices was associated with a higher serum NT-proBNP level, with a cut-off value of >101 pg/mL (sensitivity, 87.60% and specificity, 72.73%; *P* < 0.001).

**Conclusions:**

NT-proBNP was found to be a marker of the presence of esophageal varices, but not a marker of progression of liver cirrhosis. In cirrhotic patients, NT-proBNP value >101 pg/mL was shown to be a valuable noninvasive parameter in predicting the presence of varices.

Cirrhotic patients are characterized by hyperdynamic circulation with increased cardiac output, heart rate and plasma volume, normal or low arterial blood pressure, and decreased systemic vascular resistance (1-3). The pathophysiology of this syndrome consists of interactions between system hemodynamic and neurohumoral parameters (4-9).

Pro-B-type natriuretic peptide (proBNP1-108) is a 108-amino acid prohormone secreted from the cardiomyocytes secondary to cardiac wall distension and stretching, and neurohumoral activation in response to ventricular volume and pressure overload (10). The presence of brain natriuretic peptide (BNP) may be regarded as counter-regulatory to the actions of the sympathetic and renin-angiotensin-aldosterone systems (RAAS). Natriuretic peptide secretion leads to natriuresis, vasodilation activation with a concomitant inhibition of the renin-angiotensin-aldosterone system and adrenergic activity, inhibition of cardiomyocyte hypertrophy, angiogenesis promotion, and delay in the activation of cardiac fibroblasts, all of which results in improved myocardial relaxation (11,12). Patients with cirrhosis had elevated levels of N-terminal pro-B-type natriuretic peptide (NT-proBNP), without signs of reduced hepatic degradation of this hormone, and elevated plasma levels of BNP in these patients correlated best with diastolic dysfunction (4,13-16). However, there was no significant relation of NT-proBNP levels to other measures of hyperdynamic circulation, such as cardiac output or systemic vascular resistance (15). A study that evaluated the severity of disease compared to plasma levels of BNP in non-alcoholic cirrhotic patients found no significant BNP level difference between patients with and without esophageal varices (17). The role of NT-proBNP in the formation of esophageal varices and the relationship between proBNP plasma concentration and presence of varices has not been established.

The aim of this study is to investigate the association between plasma concentrations of NT-proBNP and formation of esophageal varices. Additionally, we investigated the association between plasma concentrations of NT-proBNP and severity of liver disease (Child-Pugh score), presence of ascites, as well as hemodynamic and neurohumoral parameters involved in modified circulatory homeostasis in patients with alcoholic liver cirrhosis.

## Patients and methods

### Patients

The study included 35 adult male and female patients diagnosed with alcoholic liver cirrhosis from urban and suburban parts of Zadarska County in Croatia treated in the General Hospital Zadar. Age range of eligible patients was 32-83 years. The diagnosis of liver cirrhosis included a complex set of typical clinical findings including relevant medical history, presenting symptoms, decreased prothrombin time, hypoalbuminemia with albumin-globulin inversion, hypergammaglobulinemia, advanced diffuse chronic hepatic lesion on abdominal ultrasound examination, or liver biopsy (9 patients).

Patients with liver cirrhosis of other etiology than alcohol and patients with suspected malignant comorbidity, advanced multiorgan disorders or infections, acute gastrointestinal bleeding, cardiac arrhythmias, ischemic or valvular heart disease, renal failure, along with those treated with pharmacological agents that potentially affect systemic circulation, such as beta-adrenergic blockers or nitrates were not included in the study. Patients treated with diuretics were included.

All patients signed an informed consent prior to the inclusion according to the approval of the ethics committee of General Hospital Zadar and following good clinical practice criteria.

### Study design

The patients were asked to fast overnight and then lie down for approximately two hours prior to having blood samples taken and system hemodynamic parameters measured. The blood samples were used to measure liver function tests, plasma renin activity, and NT-proBNP concentrations. System hemodynamic parameters were measured by using a sphygmomanometer, electrocardiography, and echocardiography. System circulation assessment included heart and great vessels within the mediastinum and was done by the Philips ATL Ultramark 7 (National Ultrasound, Duluth, GA, USA) ultrasound device by combining pulse Doppler and two-dimensional echocardiography of the left ventricle in lying supine patients.

### System hemodynamic parameters

We measured the serum levels of NT-proBNP, noradrenalin (NRA), and plasma rennin activity (PRA), and cardiac index (CI), systemic vascular resistance (SVR), stroke volume (SV), cardiac output (CO), and mean arterial blood pressure (MAP). Arterial blood pressure was measured with a sphygmomanometer. Mean arterial blood pressure was calculated as the sum of one-third of the systolic plus 2-fold diastolic values (MAP = 1 (systolic)/3 + 2 (diastolic)/3). The ratio of cardiac output to body surface represented the cardiac index (CI = CO/body surface). Systemic vascular resistance index (SVRI) was calculated by multiplication of MAP and 80, divided by cardiac index (SVRI = MAP × 80/CI).

Sample volume values were measured just above the aortal valve with pulsating Doppler. Aortal valve diameter values were obtained from parasternal longitudinal position and aortic valve surface area was calculated by the formula r^2^π. Stroke volume was calculated by multiplying valve surface area with the integral of valve flow velocity. Cardiac minute output volume represented multiplication of stroke volume with pulse rate recorded through synchronized electrocardiography.

### Laboratory analysis

Propeptide of the brain natriuretic peptide was analyzed in plasma by using an electrochemiluminiscence sandwich immunoassay (Elecsys proBNP, Roche Diagnostics, Meylan, France) (18). Plasma rennin activity was measured by using a commercial RIA kit (Alpco Diagnostics, Salem, NH, USA).

### Statistical analyses

According to Smirnov-Kolmogorov test, appropriate parametric tests were used in the analyses: independent *t* test, ANOVA, and Pearson correlation. Receiver operating characteristic (ROC) curve analysis was used to define cut-off value of proBNP regarding varices. *P* values less than 0.05 were considered significant. IBM SPSS, version 19.0.0.1 (SPSS Inc., Chicago, IL, USA) and MedCalc for Windows, version 11.5 (*www.medcalc.be*) were used.

## Results

This study included 35 patients with an age range from 32 to 83 years (median value, 62). Patients were divided into three groups according to the Child-Pugh classification: grade A (n = 11, 32%), B (n = 12, 34%), and C (n = 12, 34%). Esophageal varices were found in 24 (69%) patients, whereas ascites was found in 21 (60%) patients.

Mean value ± standard deviation of CI was 3.6 ± 1.0 L/min/m^2^, of SVR 2187.6 ± 625.9 dyn · s/cm^5^, of SV 85.3 ± 19.6 mL, of CO 7.1 ± 2.0 L/min, and of MAP 92.1 ± 8.1 mm Hg.

There was no significant association of NT-proBNP, NRA, and PRA with CI, SVR, and SV in neither of the groups. A significant association was found between NRA and SV and between NRA and CO (*P* = 0.053) in the group A (*P* = 0.006). In the group B, there was a negative association between NRA and MAP (*P* = 0.071). Patients without esophageal varices had a significant association between NRA and SV (*P* = 0.006), and between NT-proBNP and MAP (*P* = 0.021).

**Figure 1:**
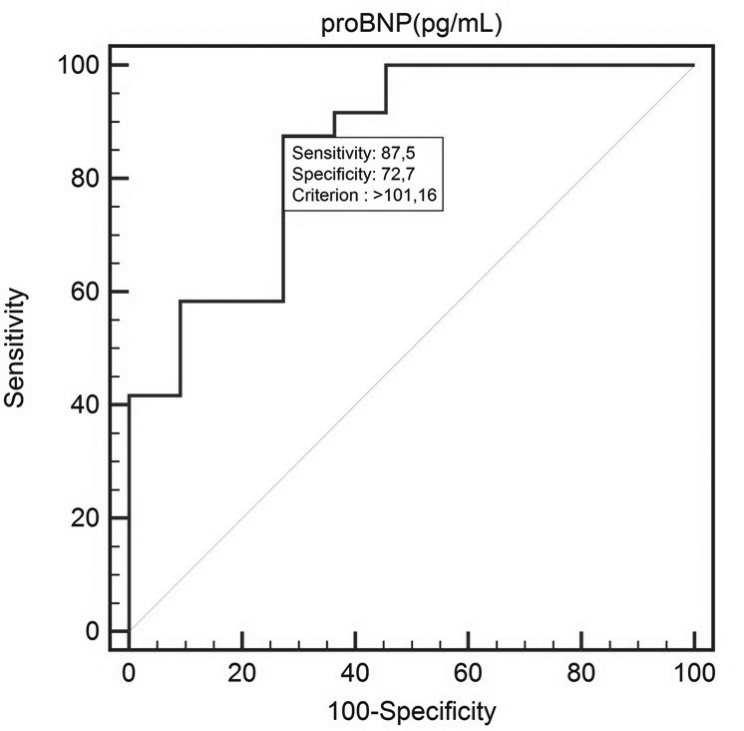
Specificity and sensitivity of -terminal pro-B-type natriuretic peptide cut-off value over 101 in the group of patients with esophageal varices. Sensitivity – 87.5%; specificity – 72.7%, criterion – 101.16 pg/mL.

Patients with esophageal varices (n = 24, 69%) had a significantly higher serum NT-proBNP level (median value 338.63 pg/mL [range 69.17-6052.5] vs 47.72 [range 11.31-570.42]; *P* = 0.014) than patients without esophageal varices. Additional analysis showed the serum level of NT-proBNP>101 pg/mL as a cut-off value for the presence of esophageal varices in patients with liver cirrhosis (*P* < 0.001), with a sensitivity of 87.60% and specificity of 72.73% (Figure 1).

Patients with liver cirrhosis also had significantly lower values of MAP (89 vs 97 mm Hg) (*P* = 0.001). There were no significant differences in NRA, PRA, CI, SVR, SV, and CO values between the groups of patients with and without esophageal varices. Patients with ascites (n = 21, 60%) had significantly lower MAP values (89 vs 96 mm Hg; *P* = 0.010) (Table 1).

**Table 1:** Average values of neurohumoral and system hemodynamic parameters

	Esophageal varices (mean ± standard deviation)		
Characteristic	present (n = 24)	not present (n = 11)	*P^†^*
proBNP (pg/mL)	1628.25 ± 1019.82	166.30 ± 127.20	0.014
NRA	827.43 ± 176.37	3.17 ± 1.49	0.316
PRA	11.56 ± 6.67	7.09 ± 5.49	0.714
CI	3.57 ± 1.09	3.73 ± 0.81	0.625
SVR	2197.14 ± 719.12	2166.85 ± 376.92	0.871
SV	82.00 ± 19.88	92.65 ± 17.79	0.128
CO	6.97 ± 2.13	7.44 ± 1.78	0.501
MAP	89.58 ± 8.03	97.75 ± 5.37	0.001
	Ascites		
	present (n = 21)	not present (n = 14)	
proBNP(pg/mL)	1700.70 ± 998.99	672.31 ± 348.73	0.127
NRA	884.50 ± 200.75	3.70 ± 2.09	0.319
PRA	7.36 ± 12.23	4.71 ± 6.43	0.409
CI	3.61 ± 1.08	3.63 ± 0.92	0.950
SVR	2146.57 ± 642.10	2249.20 ± 619.28	0.640
SV	82.43 ± 19.54	89.72 ± 19.67	0.291
CO	7.11 ± 2.10	7.13 ± 1.95	0.976
MAP	89.60 ± 9.23	95.97 ± 4.21	0.010

*Abbreviations: NRA – noradrenalin; PRA – plasma renin activity; CI – cardiac index; SVR – systemic vascular resistance; SV – stroke volume; CO – cardiac output; MAP – mean arterial pressure.

†Independent *t* test.

## Discussion

Our study showed an association between NT-proBNP and cardiac dysfunction, but the association with the progression of the disease was not established. With increasing disease stage, plasma NT-proBNP levels were greater and SV was lower. This correlates with the hyperdinamic circulation theory based on a central hypovolemia (17) and systolic dysfunction (19). Among system hemodynamic parameters, MAP was shown as a possible marker of disease progression, with a decrease in its level observed in advanced stages of liver disease, and a positive association with NT-proBNP and negative association with NRA. In advanced cirrhosis with pronounced vasodilatation, central hypovolemia and arterial hypotension, RAAS and sympathic system are highly activated causing hyperdinamic circulation. Low MAP is a result of reduced vascular reactivity to adrenaline and angiotensin-II because of increased release of nitric oxide (20).

Patients with cirrhosis and ascites present with lower blood pressure, lower peripheral vascular resistance, and increased stroke volume (21). The association between NT-proBNP and ascites is currently deemed controversial. Woo et al did not find differences in plasma NT-proBNP level between pre-ascitic and ascitic patients (19). However, Yilidizz et al found higher plasma NT-proBNP level in ascitic patients (17). Our study did not find any difference in plasma NT-proBNP level between patients with and without ascites. In our opinion, these results can be explained by high sodium intake and concomitant use of diuretic therapy, which lower BNP concentration (22).

It has been demonstrated that patients without esophageal varices have a lower cardiac index and systolic volume, higher values of peripheral vascular resistance, and mean arterial blood pressure (23). In cirrhotic patients, portal hypertension evolves due to increased portal venous resistance together with increased blood flow within the portal venous system. Both are the consequence of splanchnic arterial vasodilation caused by endotoxemia, through the opening of portosystemic collaterals, which leads to reduced activity of RAAS and intestinal disturbance resulting in increased synthesis of vasodilatators. As cirrhosis progresses, effective hypovolemia and arterial hypotension progress and RAAS induces low renal perfusion, glomerular filtration rate, and subsequently sodium and water retention, which together aggravate systolic and diastolic dysfunction, leading to increased production of NT-proBNP. NT-proBNP as a contraregulatory mechanism for decreasing portal hypertension induces natriuresis, vasodilation activation with a concomitant inhibition of the renin-angiotensin-aldosterone system and adrenergic activity. In our study, higher variceal grade was accompanied by lower levels of MAP and higher NT-proBNP plasma levels, which confirms the presented theory. We found that the cut-off of NT-proBNP serum level >101 pg/mL was a marker of esophageal varices. This could be useful as a clinical parameter and diagnostic tool for detecting esophageal varices in cirrhotic patients. However, a major limitation of our study is the small number of patients and further larger prospective studies are needed to confirm our results and establish the eventual use of this parameter in clinical practice. The small number of patients and concomitant use of diuretics could also be reasons why no association between NT-proBNP levels and stage of disease progression was found. Therefore, we can conclude that NT-proBNP could be considered as a marker of presence of varices in liver cirrhosis, but not as a marker of disease progression.

## References

[1] Schrier RW, Arroyo V, Bernardi M, Epstein M, Henriksen JH, Rodes J (1988). Peripheral artery vasodilatation hypotheses: a proposal for the initiation of renal sodium and water retention in cirrhosis.. Hepatology.

[2] Moller S, Henriksen JH (1997). Circulatory abnormalities in cirrhosis with focus on neurohumoral aspects.. Semin Nephrol.

[3] Naschitz JE, Slobodin G, Lewis RJ, Zuckerman E, Yeshurun D (2000). Heart diseases affecting the liver and liver diseases affecting the heart.. Am Heart J.

[4] Ljubičić N, Duvnjak M, Rotkvić I, Kopjar B (1990). Influence of the degree of liver failure on portal blood flow in patients with liver cirrhosis.. Scand J Gastroenterol.

[5] Padillo J, Rioja P, Munoz-Villanueva MC, Vallejo JA, Ciria R, Muntane J (2010). BNP as a marker of heart dysfunction in patients with liver cirrhosis.. Eur J Gastroenterol Hepatol.

[6] Rockey DC (1997). The cellular pathogenesis of portal hypertension: stellate cell contractility, endothelin, and nitric oxide.. Hepatology.

[7] Moller S, Henriksen JH (1998). Neurohormonal fluid regulation in chronic liver disease.. Scand J Clin Lab Invest.

[8] Henriksen JH, Moller S, Ring-Larsen H, Christensen NJ (1998). The sympathetic nervous system in liver disease.. J Hepatol.

[9] Kuntzen C, Gülberg V, Gerbes AL (2005). Use of a mixed endothelin receptor antagonist in portopulmonary hypertension: a safe and effective therapy?. Gastroenterology.

[10] Sudoh T, Maekawa K, Kojima M, Minamino N, Kangawa K, Matsuo H (1989). Cloning and sequence analysis of cDNA encoding a precursor for human brain natriuretic peptide.. Biochem Biophys Res Commun.

[11] Mukoyama M, Nakao K, Saito Y, Ogawa Y, Hosoda K, Suga S (1990). Human brain natriuretic peptide, a novel cardiac hormone.. Lancet.

[12] Cheung BMY, Kumana CR (1998). Natriuretic peptides-relevance in cardiac disease.. JAMA.

[13] Komeichi H, Moreau R, Cailmail S, Gaudin C, Lebrec D (1995). Blunted natriuresis and abnormal systemic haemodynamic responses to C-type and brain natriuretic peptides in rats with cirrhosis.. J Hepatol.

[14] Salo J, Jimenez W, Kuhn M, Gines A, Gines P, Fernandez-Esparrach G (1996). Urinary excretion of urodilatin in patients with cirrhosis.. Hepatology.

[15] Henriksen JH, Gotze JP, Fuglsang S, Christensen E, Bendtsen F, Moller S (2003). Increased circulating pro-brain natriuretic peptide and brain natriuretic peptide in patients with cirrhosis: relation to cardiovascular dysfunction and severity of disease.. Gut.

[16] Wong F, Siu S, Liu P, Blendis LM (2001). Brain natriuretic peptide: is it a predictor of cardiomyopathy in cirrhosis?. Clin Sci.

[17] Yildiz R, Yildirim B, Karincaoglu M, Harputluoglu M, Hilmioglu F (2005). Brain natriuretic peptide and severity of disease in non-alcoholic cirrhotic patients.. J Gastroenterol Hepatol.

[18] Hess G, Runkel S, Zdunek D, Hitzler WE (2005). Reference interval determination for N:terminal.B-type natriuretic peptide (NT-proBNP): a study in blood donors.. Clin Chim Acta.

[19] Woo JJ, Koh YY, Kim HJ, Chung JW, Chang KS, Hong SP (2008). N-terminal pro B-type natriuretic peptide and the evaluation of cardiac dysfunction and severity of disease in cirrhotic patients.. Yonsei Med J.

[20] Hennenberg M, Trebicka J, Sauerbruch T, Heller J (2008). Mechanisms of extrahepatic vasodilation in portal hypertension.. Gut.

[21] Acosta F, Sansano T, Palenciano CG, Domenech P, Falcon L, Robles R (2005). Differential response of the systemic and pulmonary circulation related to disease severity of cirrhosis.. Transplant Proc.

[22] de Lemos JA, McGuire DK, Drazner MH (2003). B-type natriuretic peptide in cardiovascular disease.. Lancet.

[23] Zekanovic D, Ljubicic N, Boban M, Nikolic M, Delic-Brkljacic D, Gacina P (2010). Doppler ultrasound of hepatic and system hemodynamics in patients with alcoholic liver cirrhosis.. Dig Dis Sci.

